# Adenocarcinoma in the Jejunum Presenting as Acute Abdomen and Melena in an Otherwise Healthy Patient: A Case Report

**DOI:** 10.7759/cureus.72011

**Published:** 2024-10-21

**Authors:** Edwin J Paredes González, Keiry M Gonzalez Benitez, Lester J Tavarez Reynoso, Leandro Tapia Garcia

**Affiliations:** 1 General Surgery, Hospital General Docente de la Policía Nacional, Santo Domingo, DOM; 2 Research/Wound Care, SerenaGroup Research Foundation, Pittsburgh, USA

**Keywords:** acute abdomen, jejunal adenocarcinoma, melena, rare disease, small bowel

## Abstract

A jejunal adenocarcinoma is a rare form of cancer that is difficult to diagnose due to its vague and non-specific symptoms, often leading to delayed treatment and poor outcomes. In this case, we report a 43-year-old male who presented with acute abdominal pain, weight loss, and melena. A CT scan revealed a mass in the small intestine, and subsequent exploratory laparotomy confirmed the presence of a tumor, which was surgically excised. Histopathological analysis confirmed jejunal adenocarcinoma with positive CDX2 staining, ruling out other adenocarcinoma subtypes. This case underscores the importance of including jejunal adenocarcinoma in differential diagnoses of acute abdomen and highlights the critical role of early surgical intervention in improving patient outcomes.

## Introduction

Jejunal adenocarcinoma is an exceptionally rare malignancy, representing less than 1% of all gastrointestinal cancers, despite the small intestine comprising approximately 75% of the overall length and 90% of the mucosal surface area of the digestive tract [1]. The relative infrequency of malignancies in the small intestine, compared to the colon and stomach, has often been attributed to the rapid transit time of intestinal contents, the protective role of the mucosal immune system, and the relatively low bacterial load in the small intestine [2]. Nevertheless, when adenocarcinoma does originate in the small bowel, especially the jejunum, it presents a significant clinical challenge due to its nonspecific and often subtle symptomatology [3].

Patients with jejunal adenocarcinoma frequently present with vague symptoms such as abdominal pain, weight loss, nausea, and gastrointestinal bleeding, which can be easily mistaken for more common conditions such as peptic ulcer disease, Crohn’s disease, or other small bowel pathologies [4]. The atypical presentation of these symptoms often leads to delayed diagnosis, with many cases not being identified until advanced staging when the disease has already metastasized [5]. This delayed diagnosis significantly impacts prognosis, with advanced-stage jejunal adenocarcinoma associated with poor outcomes and limited therapeutic options [3].

In this report, we present the case of a 43-year-old male patient who presented to the Hospital General Docente de la Policia Nacional (HOSGEDOPOL), in Santo Domingo, Dominican Republic, with an acute abdomen. His clinical presentation and the subsequent diagnostic workup highlight the challenges associated with diagnosing this rare and insidious malignancy. Early and accurate diagnosis is crucial for improving the chances of curative surgical intervention and overall patient outcomes [2,3]. This article was previously presented as a poster at the 2024 Dominican College Of Surgeons on June 27-30, 2023.

Through this case report, we aim to raise awareness of the critical importance of considering jejunal adenocarcinoma in the differential diagnosis of acute abdomen and other unexplained gastrointestinal symptoms and to emphasize the need for timely intervention. Furthermore, we expect to contribute valuable insights into this rare malignancy and discuss potential approaches for its management, which could guide future therapeutic strategies.

## Case presentation

**Figure 1:**
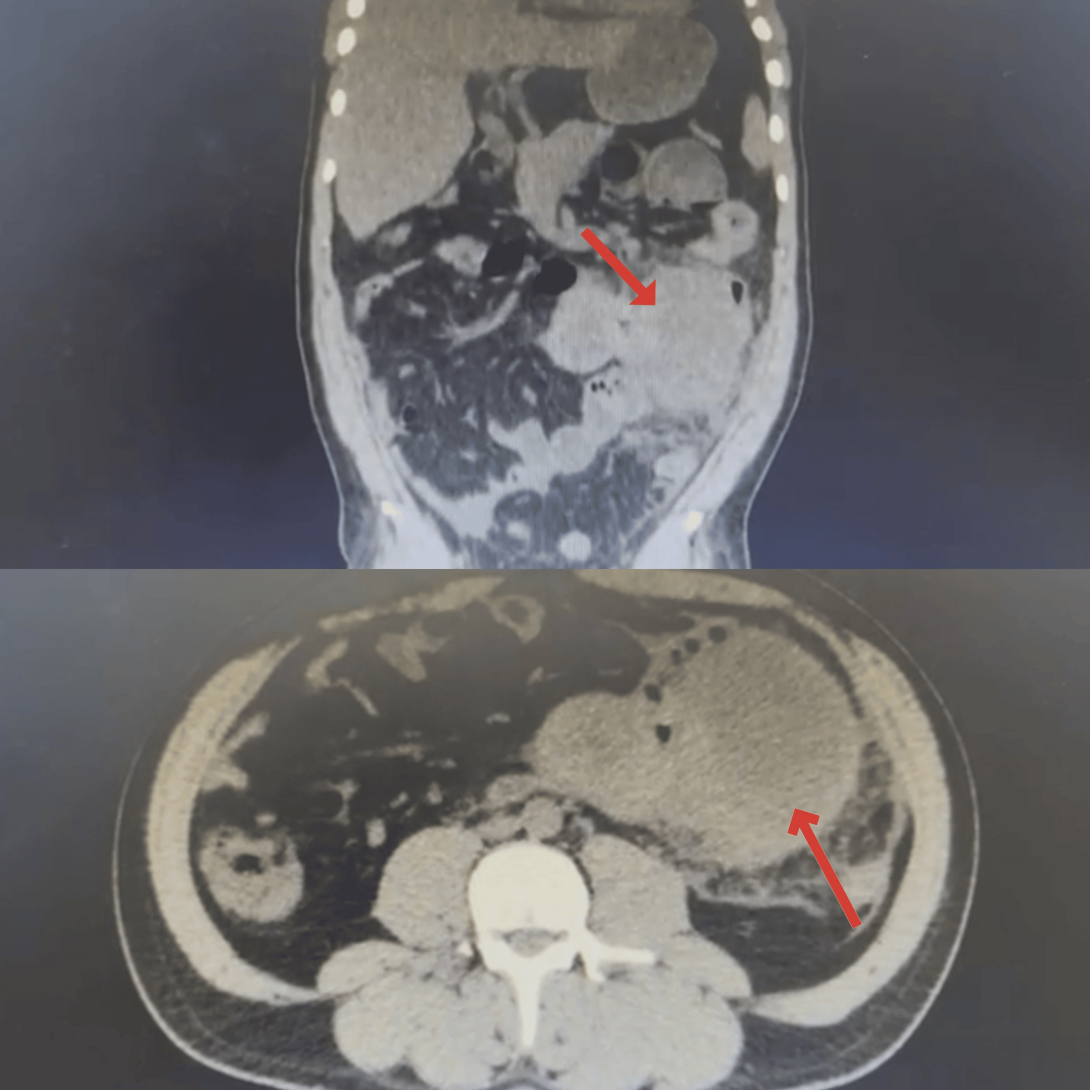
Coronal (upper image) and axial (bottom image) CT of the patient The CT of the patient showed an enlarged tumor of the small intestine (indicated by the arrows).

A 43-year-old mulatto male, with no known prior medical history, presented to the surgery department at HOSGEDOPOL with acute abdominal pain. The patient reported a three-month history of progressive symptoms, including a 10-pound weight loss, colicky pain localized to the left flank, and the passage of dark, tarry stools consistent with melena. Three days before his presentation, his symptoms were exacerbated with more intense abdominal pain, persistent nausea progressing to bilious vomiting, and four episodes of liquid stools. Given these symptoms, the initial diagnostic and laboratory workup (Table 1) included abdominal radiography and computed tomography (CT) scan with intravenous contrast (Figure 1), which revealed a suspicious mass in the small intestine that was not palpable on the physical exam and splenomegaly.

**Table 1:** Comprehensive laboratory report upon admission RBC, Red blood cells; WBC, White blood cells; HGB, Hemoglobin; HCT, Hematocrit; MCV, Mean corpuscular volume; MCH, Mean corpuscular hemoglobin; МСНС, Mean corpuscular hemoglobin concentration; RDW-SD, Red cell distribution width - standard deviation; RDW-CV, Red cell distribution width - coefficient of variation; PLT, Platelets; MPV, Mean platelet volume; PDW, Platelet distribution width; РСТ, Procalcitonin; UREA, Urea; BUN, Blood urea nitrogen; CLORO, Chlorine; SODIO, Sodium; POTASIO, Potassium; HIV, Human immunodeficiency virus; VDRL, Venereal Disease Research Laboratory; HEPATITIS B (HBsAg), Hepatitis B (hepatitis B surface antigen)

Parameter	Result	Reference Range	Unit	Notes
WBC	20.04 H	4.0–10.0	10³/μL	High
Lymph#	0.54 L	1.0–5.0	10³/μL	Low
Mon#	0.68	0.1–1.0	10³/μL	Normal
Neu#	18.72 H	1.5–7.0	10³/μL	High
Eos#	0.02	0.02–0.50	10³/μL	Normal
Bas#	0.08	0.00–0.10	10³/μL	Normal
Lymph%	2.7 L	25.0–50.0	%	Low
Mon%	3.4	2.0–10.0	%	Normal
Neu%	93.4 H	50.0–72.0	%	High
Eos%	0.1 L	0.5–5.0	%	Low
Bas%	0.4	0.0–1.0	%	Normal
RBC	4	4.0–6.20	10⁶/μL	Normal
HGB	7.1 L	11.0–17.0	g/dL	Low
HCT	24.4 L	35.0–55.0	%	Low
MCV	60.9 L	80.0–100.0	fL	Low
MCH	17.8 L	26.0–34.0	pg	Low
MCHC	29.2 L	31.0–35.5	g/dL	Low
RDW-SD	32.1 L	35.0–56.0	fL	Low
RDW-CV	19.5 H	11.0–16.0	%	High
PLT	425	150–450	10³/μL	Normal
MPV	4.5 L	7.0–11.0	fL	Low
PDW	18.5 H	10.0–18.0	%	Slightly High
PCT	0.19 L	0.200–0.500	%	Low
Urea	50.5 H	15–45	mg/dL	High
BUN	23.6 H	7–21	mg/dL	High
Creatinine	4.3 H	0.6–1.1 (female) / 0.7–1.4 (male)	mg/dL	High
CLORO	99.2	98–107	mEq/L	Normal
SODIO	133.0 L	135–165	mEq/L	Low
POTASIO	4.5	3.6–5.5	Mmol/L	Normal
HIV	Negative	Negative		
VDRL	Non-reactive	Non-reactive		
Hepatitis B (HBsAg)	Negative	Negative		
Hepatitis C (HCV)	Negative	Negative		

**Figure 2:**
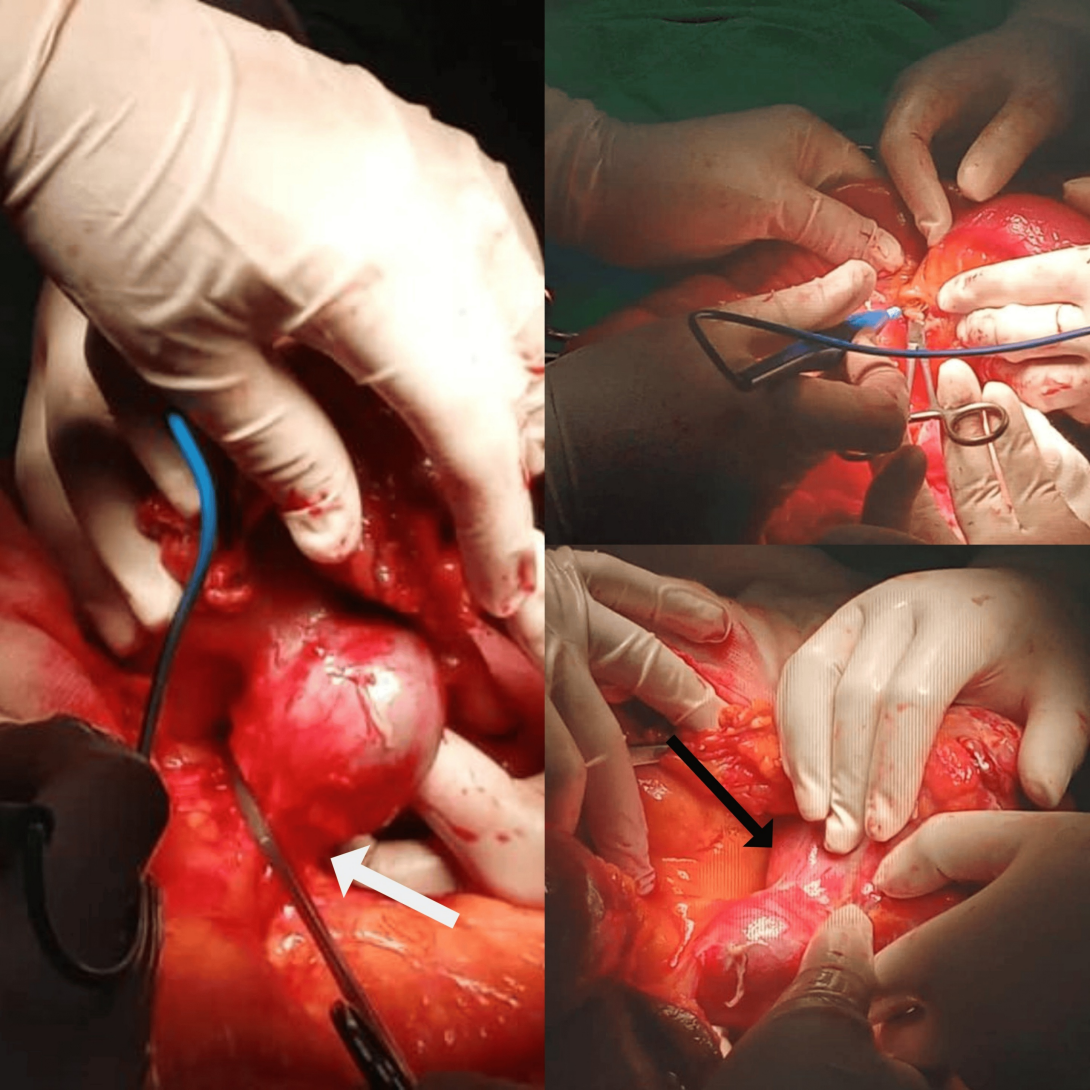
Excision of the mass in the operating room The tumor adhered to the anterior abdominal wall (black arrow) and the mesentery (white arrow).

**Figure 3:**
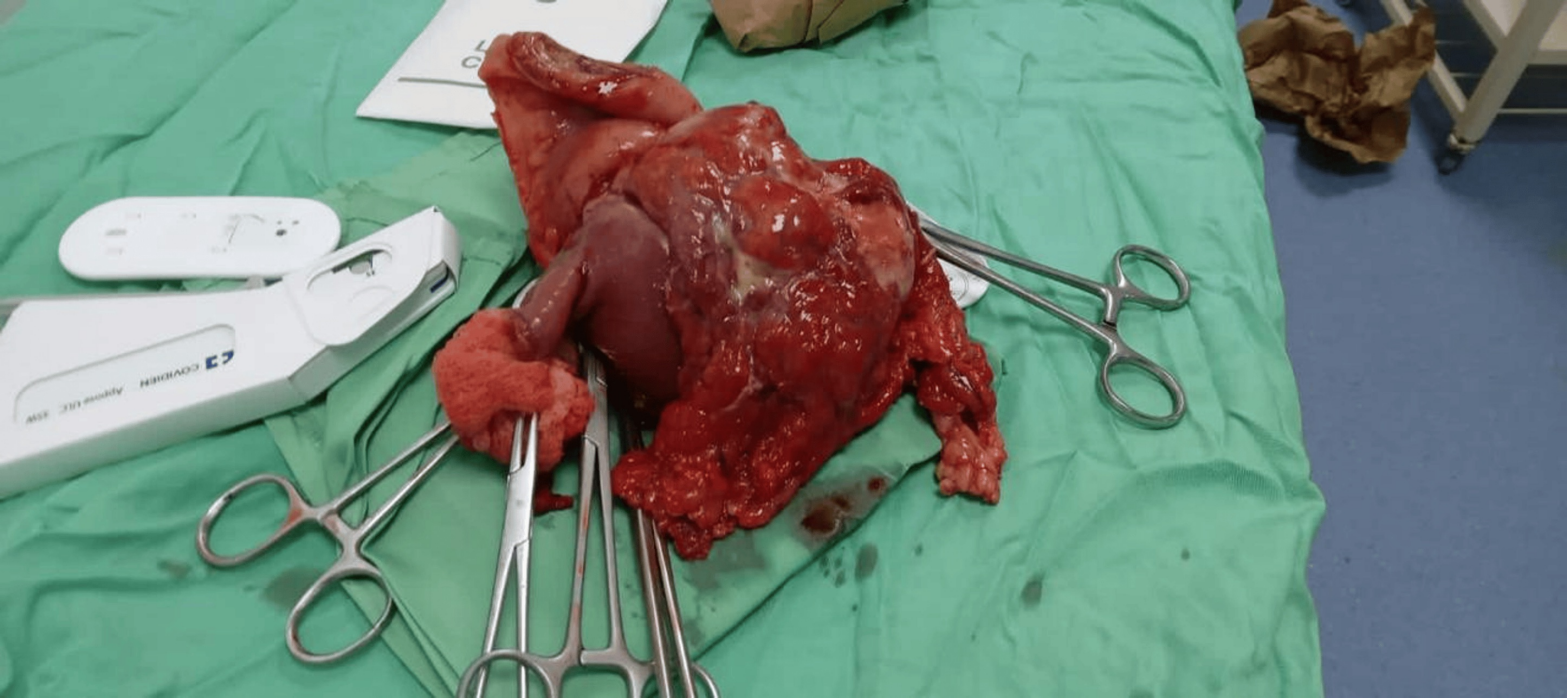
Surgical resection including the tumor within the jejunum

Considering the imaging findings and the patient’s clinical presentation, an exploratory laparotomy was performed. During the surgery, a 20 cm tumor was identified 30 cm from the Treitz ligament (Figure 2). The mass was adherent to the abdominal wall, the anterior surface of the descending colon, and the root of the mesentery, necessitating careful enterocolonic dissection, partial mesentectomy, and resection of 25 cm jejunum with 5 cm of oncological margins classified as R0 resection and anastomosis, and 12 lymph nodes were resected and were positive. The excised specimen (Figure 3) was sent for histopathological examination, and immunohistochemical analysis was conducted to further characterize the tumor.

The immunohistochemical analysis revealed that the tumor was positive for CDX2, consistent with a gastrointestinal origin; the specimen was negative for CK7, CK20, and PAX-8, ruling out other common adenocarcinoma subtypes. Based on these findings, the patient was diagnosed with jejunal adenocarcinoma with a T4N3M0 classification, one of the rarest forms of small bowel cancers. Post-operatively, the patient did not experience any complications and was closely monitored with routine follow-ups, lab work, and imaging studies, with an improved survival prognosis secondary to the early and successful surgical intervention. After seven days of the surgery, the patient was discharged in stable conditions from the hospital.

## Discussion

In this case, the patient presented with a three-month history of weight loss, colicky abdominal pain, and melena, all of which are non-specific symptoms that are the usual presentation of small bowel tumors. Imaging studies, particularly computed tomography (CT), played a critical role in identifying the jejunal mass which was visualized during exploratory laparotomy [6]. Studies have shown that CT scans have high sensitivity and specificity in detecting small bowel tumors, especially when combined with other diagnostic modalities such as enteroclysis or magnetic resonance imaging (MRI) [3]. Endoscopy, though limited in reach for the small intestine, can also be useful when paired with capsule endoscopy, providing better visualization of the small bowel mucosa in suspected cases of small bowel tumors [7]. The surgical findings in this case, including the tumor's adherence to the abdominal wall, descending colon, and mesentery, further emphasize the aggressive nature of jejunal adenocarcinoma [8]. It is also worth noting that these types of rare tumors usually present themselves as obstruction but this report shows us that they can also appear as melena, which is not typical for those malignancies [9].

The immunohistochemical analysis was essential in confirming the diagnosis, with the tumor showing positive staining for CDX2 and negative for CK7, CK20, and PAX-8. CDX2 is a reliable marker for gastrointestinal origin, particularly in distinguishing primary small bowel adenocarcinoma from metastatic adenocarcinomas of other origins [10]. The absence of positive CK7 and CK20 staining, which are typically associated with adenocarcinomas of the lung, breast, and colorectum, respectively, further supported the diagnosis of primary jejunal adenocarcinoma. This immunohistology profile is consistent with previous studies that emphasize the utility of these markers in differentiating small bowel adenocarcinoma from other malignancies [11,12]. Additionally, the absence of the rare PAX-8 positive stain, in this case, ruled out metastatic involvement from a Müllerian or renal origin, which contributes to more complex and poor prognostic diagnoses [13].

The prognosis for jejunal adenocarcinoma remains poor, particularly when diagnosed at an advanced stage. The five-year survival rates are highly dependent on the staging at diagnosis. Early-stage (Stage I) tumors have a survival rate of 50-60%, while Stage IV tumors have a survival rate as low as 3-5% [14]. Surgical resection remains the cornerstone of treatment, with the goal of achieving an R0 resection (complete removal of the tumor with negative margins) to improve survival outcomes [15]. This case demonstrates not only the importance of early detection but also the need for comprehensive diagnostic strategies that include advanced imaging and immunohistochemical analysis. The role of adjuvant chemotherapy, particularly in cases with lymph node involvement or positive margins, requires further exploration due to the limited data available from large-scale studies [1]. Clinicians must maintain a high index of suspicion for small bowel malignancies in patients with unexplained gastrointestinal symptoms to ensure timely and effective treatment.

## Conclusions

Jejunal adenocarcinoma, though rare, should be considered in the differential diagnosis of patients presenting with nonspecific gastrointestinal symptoms such as unexplained abdominal pain, weight loss, and gastrointestinal bleeding. This case also enlightens us that it could present as subtle as melena. Early detection remains challenging due to the subtlety of symptoms, which often leads to a delayed diagnosis and a poor prognosis. However, prompt surgical intervention, as demonstrated in this case, can significantly improve outcomes. Future research should focus on improving diagnostic strategies and exploring adjuvant therapies to enhance survival in patients with this rare malignancy.
